# Ultra-sensitive determination of carcinogenic aflatoxins in food matrices using a novel magnetic bimetallic ZIF-8@chitosan sorbent and dispersive micro-solid phase extraction

**DOI:** 10.1039/d5ra09926a

**Published:** 2026-05-26

**Authors:** Alireza Shams, Foroughalzaman Kazempoor Mofrad, Mahdi Ghorbani

**Affiliations:** a Department of Chemistry, Ma. C., Islamic Azad University Mashhad Iran farsh1358@yahoo.com kazempoormofradforough@gmail.com; b Department of Chemistry, Faculty of Sciences, Ferdowsi University of Mashhad Mashhad Iran ghorbani267@yahoo.com ghorbani267@mail.um.ac.ir

## Abstract

A dispersive micro-solid phase extraction (D-µ-SPE) method coupled with HPLC-FLD was developed for the sensitive determination of carcinogenic aflatoxins. A novel magnetic composite sorbent was synthesized by integrating bimetallic Co/Zn ZIF-8, chitosan, and Fe_3_O_4_ nanoparticles. Statistical comparison revealed that bimetallic Co/Zn ZIF-8 exhibited significantly enhanced aflatoxin extraction efficiency over its monometallic counterpart, with subsequent functionalization using chitosan and magnetization further improving performance. Key extraction parameters were systematically optimized using experimental design. The validated method demonstrated wide linearity (0.02–120 ng mL^−1^; *R*^2^ > 0.993), and low detection limits (LODs: 0.006–0.07 ng mL^−1^), and quantification limits (LOQs: 0.02–0.21 ng mL^−1^). Precision ranged from 3.7–4.7% (intra-day) to 4.5–6.2% (inter-day). Given the critical need to monitor these potent toxins in high-consumption and high-risk commodities, the method was successfully applied to commercial mineral water (potential environmental contamination), rice (global dietary staple), and peanut (notoriously susceptible crop) samples. Relative recoveries of 91.2–98.0% (RSD < 4.4%) confirmed the method's robustness and accuracy for complex matrices.

## Abstract 2

A novel magnetic bimetallic Co/Zn ZIF-8@chitosan sorbent enables ultra-sensitive extraction of carcinogenic aflatoxins from food matrices with exceptional recovery and low detection limits.

## Graphical abstract

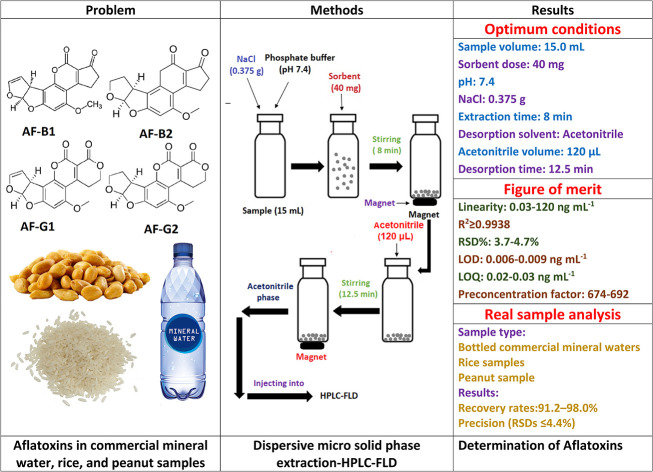

## 1 Introduction

Aflatoxins, which are highly carcinogenic secondary metabolites predominantly biosynthesized by the fungal species *Aspergillus flavus* and *Aspergillus parasiticus*, pose a pervasive global threat to food safety, public health, and agricultural economies, thus demanding rigorous scientific investigation.^1,2^ Contamination of critical staple crops, including maize, groundnuts, tree nuts, spices, and oilseeds, occurs both pre- and post-harvest, particularly under warm, humid conditions that are increasingly prevalent as a result of climate change.^3,4^ This widespread contamination leads to significant human exposure through the food chain.^5^ The human health consequences are severe and multifaceted: chronic ingestion of low levels, particularly aflatoxin B1, is a well-established primary risk factor for hepatocellular carcinoma, while acute exposure can induce fatal aflatoxicosis characterized by hemorrhagic necrosis of the liver.^6^ Beyond their potent carcinogenicity, aflatoxins exhibit significant immunosuppressive, mutagenic, and teratogenic effects, contributing to childhood growth impairment, increased susceptibility to infectious diseases such as HIV/AIDS and malaria, and substantial morbidity and mortality, especially in developing regions with limited regulatory infrastructure.^7–9^ Consequently, the accurate determination and quantification of aflatoxins are paramount for robust risk assessment, effective regulatory enforcement, and the development of targeted mitigation strategies. High-Performance Liquid Chromatography (HPLC), frequently coupled with fluorescence detection (FLD) or mass spectrometry (MS), serves as the benchmark analytical technique for this purpose, offering the requisite high sensitivity for detection at stringent regulatory limits (*e.g.*, low parts per billion) mandated by international bodies (*e.g.*, EU, FDA, Codex Alimentarius) and exceptional selectivity for the reliable separation and quantification of individual aflatoxins (B1, B2, G1, G2, M1) within complex food and feed matrices.^10,11^

However, the inherent complexity of these matrices presents a significant analytical challenge, necessitating efficient and reliable sample preparation prior to HPLC analysis to ensure accuracy and sensitivity.^12,13^ Co-extracted compounds such as lipids, proteins, pigments, carbohydrates, and other interferents can induce detrimental matrix effects, cause column fouling, interfere with detection, or mask target analytes, thereby compromising the reliability of quantification.^14^ Robust sample preparation is therefore essential to isolate, concentrate, and purify aflatoxins from complex backgrounds.^15^ Among available techniques, dispersive micro solid-phase extraction (D-µ-SPE) has emerged as a highly advantageous approach for aflatoxin analysis.^16^ D-µ-SPE involves the direct dispersion of a small quantity of solid sorbent material into the sample extract, facilitating rapid and efficient adsorption of target analytes.^17^ This method offers substantial benefits over conventional solid-phase extraction (SPE), including drastically reduced solvent consumption (aligning with green analytical chemistry principles), shorter processing times, simplified procedures that minimizing manual handling, and lower operational costs.^16,18^ Crucially, the versatility of D-µ-SPE allows for strategic selection of the sorbent material to optimize selectivity and enrichment factors for aflatoxins, even at ultra-trace levels, thereby enhancing subsequent HPLC performance by lowering detection limits, improving quantification accuracy, increasing method robustness, and extending column lifetime.^19,20^

The efficacy of D-µ-SPE is fundamentally dependent on the performance characteristics of the sorbent material, which acts as the active interface for the selective capture and pre-concentration of target aflatoxins while simultaneously excluding matrix interferents.^16,21^ Key parameters such as sorption capacity, selectivity, sorption/desorption kinetics, and reusability are dictated by the sorbent's chemical composition, surface area, pore structure, and surface functionality.^22,23^ In this context, Metal–Organic Frameworks (MOFs) – crystalline porous materials constructed from metal ions or clusters coordinated to multidentate organic linkers – represent a revolutionary class of sorbents for D-µ-SPE.^24^ Their exceptional suitability arises from their ultra-high surface areas and porosity, enabling significant analyte pre-concentration, coupled with unparalleled tunability of pore size and surface functionality to enhance affinity and selectivity for specific aflatoxins through tailored interactions (*e.g.*, π–π stacking, hydrogen bonding, hydrophobic effects).^25,26^ Bimetallic MOFs, incorporating two distinct metal ions within a single framework, offer further enhanced performance as D-µ-SPE sorbents.^27,28^ The synergistic interaction between the dual metals modulates properties such as Lewis acidity, redox potential, and pore chemistry, leading to improved binding affinity, selectivity, and chemical stability compared to monometallic analogs.^29,30^ This design flexibility allows for precise engineering of active sites optimized for strong interaction with aflatoxin molecules, thereby boosting extraction efficiency and matrix tolerance. Furthermore, functionalization of bimetallic MOFs with magnetic cores (*e.g.*, Fe_3_O_4_ nanoparticles) enables rapid and convenient magnetic separation after extraction, significantly streamlining the D-µ-SPE workflow.^31,32^

## 2 Experimental

### 2.1. Materials

Aflatoxin Mix 4 solution (containing B_1_, B_2_, G_1_, and G_2_ at 20 µg mL^−1^ each in acetonitrile; Sigma-Aldrich, USA) was used as analytical standards. Zinc(ii) nitrate hexahydrate (99.0%), cobalt(ii) nitrate hexahydrate (≥99.0%), 2-methylimidazole (99.0%), sodium tripolyphosphate (≥98.0%), sodium dihydrogen phosphate (≥99.0%), disodium hydrogen phosphate (≥99.0%), formic acid (≥95.0%), and low molecular weight chitosan were sourced from Sigma-Aldrich (USA). Fe_3_O_4_ nanoparticles (15–20 nm diameter; US Research Nanomaterials, Inc., USA) were used as received. All solvents—methanol, ethanol, isopropanol, acetone, and acetonitrile (HPLC grade)—were obtained from Merck (Germany).

### 2.2. Instruments

Aflatoxins B_1_, B_2_, G_1_, and G_2_ were quantified using high-performance liquid chromatography (HPLC; Knauer, Germany) coupled with post-column photochemical derivatization (UVE photoreactor; LC Tech, Germany) and fluorescence detection (RF-20A detector; Shimadzu, Japan). This derivatization system operates without chemical reagents, relying on UV irradiation to convert aflatoxins B_1_ and G_1_ into their more fluorescent derivatives (B_2_a and G_2_a), thereby enhancing detection sensitivity. Aflatoxins B_2_ and G_2_ are naturally fluorescent and do not require derivatization, allowing simultaneous determination of all four analytes in a single chromatographic run. Fluorescence detection utilized excitation and emission wavelengths of 362 nm and 435 nm, respectively. Chromatographic separation was achieved using a Eurospher 100-5 C18 column (250 mm × 4.6 mm i.d., 5 µm particle size; Knauer, Germany). The mobile phase consisted of methanol/acetonitrile (50 : 50, v/v) and 0.05 mM phosphate buffer (pH 4.0), delivered at a ratio of 40 : 60 (v/v) and a flow rate of 1.0 mL min^−1^. The injection volume was 20.0 µL, and total run time was 15 min per sample. The retention times were approximately 5.8 min for AFG_1_, 7.2 min for AFB_1_, 9.1 min for AFG_2_, and 11.3 min for AFB_2_. The synthesized sorbent material was characterized using Fourier transform infrared spectroscopy (FT-IR; Bruker Tensor series, Germany) and field-emission scanning electron microscopy (FE-SEM; Mira 3 Tescan; Czech Republic). Sample pH adjustments were performed using a calibrated pH meter (Metrohm 780, Switzerland).

### 2.3. Sorbent preparation

#### 2.3.1. Bimetallic Co/Zn ZIF-8

A methanolic solution (10 mL) containing zinc nitrate hexahydrate (1.875 mmol) and cobalt(ii) nitrate hexahydrate (0.469 mmol) was prepared. Separately, 2-methylimidazole (7.5 mmol) was dissolved in methanol (15 mL). Both solutions were sonicated for 5 minutes to ensure complete dissolution. Subsequently, the metal salt solution was added incrementally to the ligand solution under continuous sonication: 5 mL was added over 3 minutes, followed by an additional 5 mL added over 10 minutes. The resulting precipitate was isolated by centrifugation, washed thoroughly with methanol, and dried under vacuum.

#### 2.3.2. Magnetic bimetallic Co/Zn ZIF-8@chitosan

Chitosan (0.5 g) was dissolved in 25 mL of aqueous acetic acid (20% w/v) with magnetic stirring for a minimum of 10 minutes. Bimetallic Co/Zn ZIF-8 (0.4 g) and magnetite (Fe_3_O_4_, 0.4 g) were dispersed into the chitosan solution by sonication for 3 minutes, yielding a homogeneous suspension. Sodium tripolyphosphate solution (TPP, 25 mL, 0.2 mg mL^−1^) was then added dropwise to the stirring suspension over 20 minutes to induce ionic cross-linking. The mixture was further sonicated for 30 minutes to complete composite formation. The resulting magnetic composite was collected by magnetic separation, washed sequentially with distilled water (twice), and dried under vacuum at 50 °C for 10 hours.

#### 2.3.3. Zn-ZIF-8

Zn ZIF-8 was synthesized following the same procedure as for the bimetallic Co/Zn ZIF-8 (Section 2.3.1), but using only zinc nitrate hexahydrate without the addition of cobalt(ii) nitrate hexahydrate.

### 2.4. Sorbent characterization

**Fig. 1:**
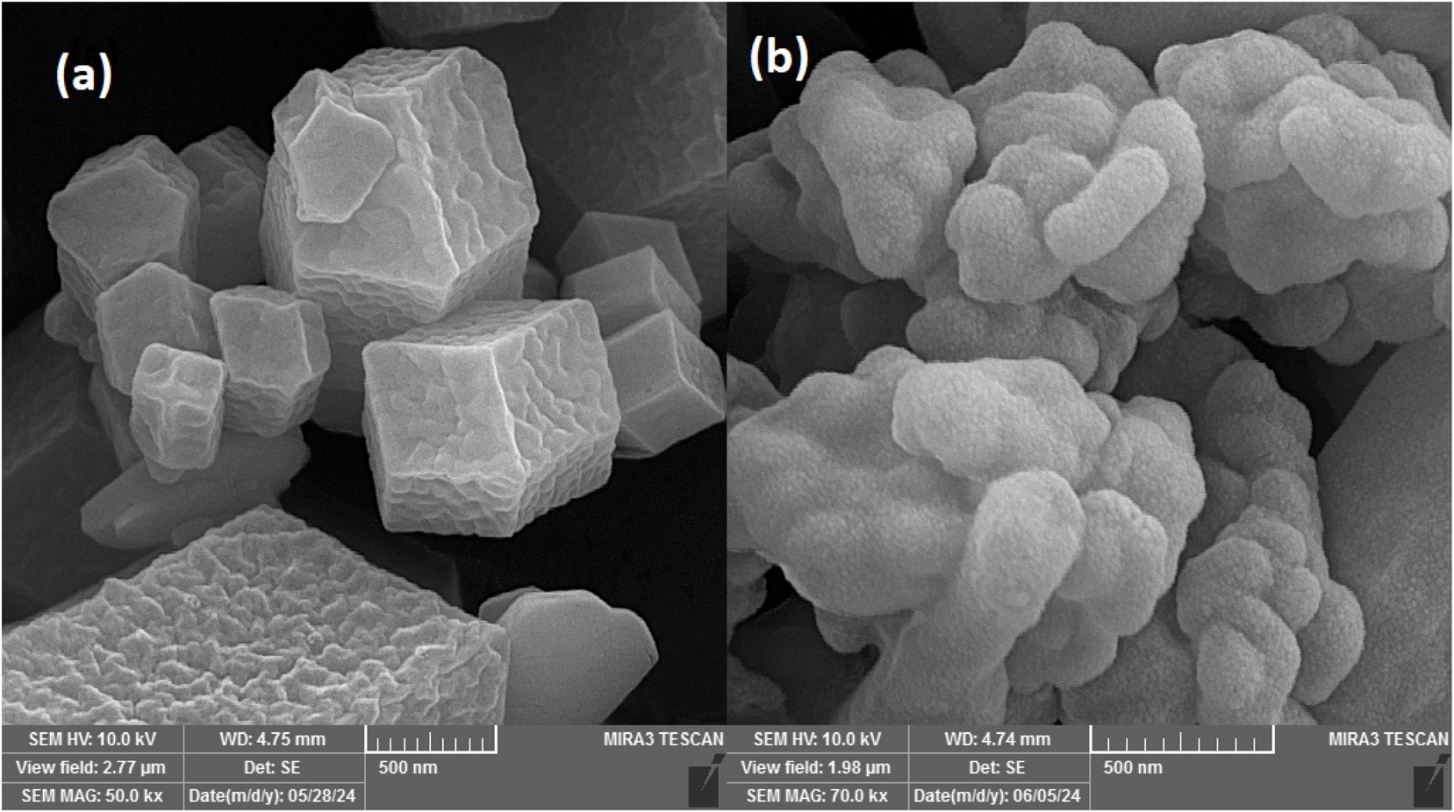
SEM images of (a) bimetallic Co/Zn ZIF-8 and (b) magnetic bimetallic Co/Zn ZIF-8@chitosan nanocomposite.

Fig. 1 presents the scanning electron microscopy (SEM) image of (a) the bimetallic Co/Zn ZIF-8 and (b) the magnetic bimetallic Co/Zn ZIF-8@chitosan nanocomposite, acquired at magnifications of 50.0 kx and 70.0 kx, respectively. The pristine bimetallic Co/Zn ZIF-8 (Fig. 1a) exhibits a well-defined rhombic dodecahedral morphology characteristic of ZIF-8 frameworks, with sharp edges and smooth facets averaging 500–600 nm in diameter, indicative of high crystallinity and phase purity. In contrast, the magnetic Co/Zn ZIF-8@chitosan composite (Fig. 1b) reveals a distinct core–shell architecture, where Fe_3_O_4_ nanoparticles (5–20 nm) are uniformly embedded within a porous chitosan matrix that conformally encapsulates the bimetallic ZIF-8 crystals. This hierarchical structure results in a roughened surface topography with increased nano- and mesoporosity (pore diameter ∼2–50 nm), attributed to chitosan's fibrillar network cross-linking the magnetic and ZIF components. The morphological evolution from crystalline polyhedra (Fig. 1a) to an interconnected porous hydrogel composite (Fig. 1b) substantially enhances aflatoxin adsorption efficacy by: (i) augmenting accessible surface area (as confirmed by BET analysis, *vide infra*), (ii) introducing multifunctional binding sites (–NH_2_, –OH) from chitosan for hydrogen bonding and electrostatic interactions with aflatoxins, and (iii) enabling magnetic separation (*via* embedded Fe_3_O_4_) for operational convenience.

**Fig. 2:**
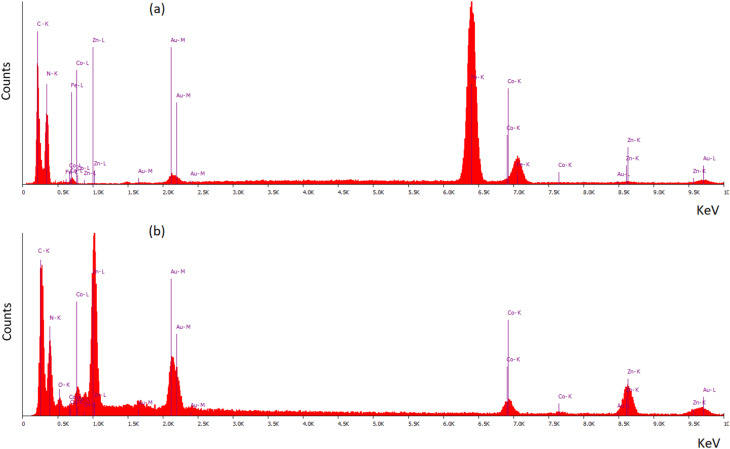
EDX patterns of (a) bimetallic Co/Zn ZIF-8 and (b) magnetic bimetallic Co/Zn ZIF-8@chitosan nanocomposite.

The EDX patterns of the bimetallic Co/Zn ZIF-8 and the magnetic bimetallic Co/Zn ZIF-8@chitosan composite are shown in Fig. 2. The EDX pattern for the bimetallic Co/Zn ZIF-8 (Fig. 2a) reveals distinct peaks corresponding to the fundamental elements of the framework. A prominent peak for Zinc (Zn) is observed, confirming the primary metal node in the ZIF-8 structure. A clear peak for Cobalt (Co) is also present, verifying the successful partial substitution of Zn^2+^ by Co^2+^ ions to form the bimetallic system. Strong peaks for Carbon (C) and Nitrogen (N) are evident, originating from the 2-methylimidazolate organic linkers that bridge the metal centers. An Oxygen (O) peak is also detected, which can be attributed to surface hydroxyl groups or adsorbed moisture. The absence of peaks for other elements confirms the purity of the synthesized bimetallic ZIF-8 material. The EDX pattern of the magnetic bimetallic Co/Zn ZIF-8@chitosan composite (Fig. 2b) exhibits the characteristic peaks of all constituent phases, demonstrating successful integration. Peaks for Zinc (Zn) and Cobalt (Co) persist, confirming the retention of the bimetallic ZIF-8 framework within the composite. Most importantly, a new and intense peak for Iron (Fe) appears, providing direct evidence for the incorporation of the Fe_3_O_4_ magnetic nanoparticles. The Carbon (C) peak remains strong, reflecting contributions from both the ZIF-8 linkers and the chitosan biopolymer matrix. The Nitrogen (N) peak is also observed, arising from the imidazolate linkers of the ZIF and the amine (–NH_2_) and amide (–NHCO–) functional groups of chitosan. The Oxygen (O) peak is significantly more intense compared to the pure ZIF-8 spectrum, consistent with the substantial oxygen content contributed by both the Fe_3_O_4_ nanoparticles (Fe_3_O_4_) and the abundant hydroxyl (–OH) and ether (–C–O–C–) groups within the chitosan polysaccharide chain. The combined presence of Fe, Co, Zn, C, N, and O, with no extraneous elemental peaks, conclusively verifies the formation of the ternary magnetic composite material comprising Fe_3_O_4_, bimetallic Co/Zn ZIF-8, and chitosan.

**Fig. 3:**
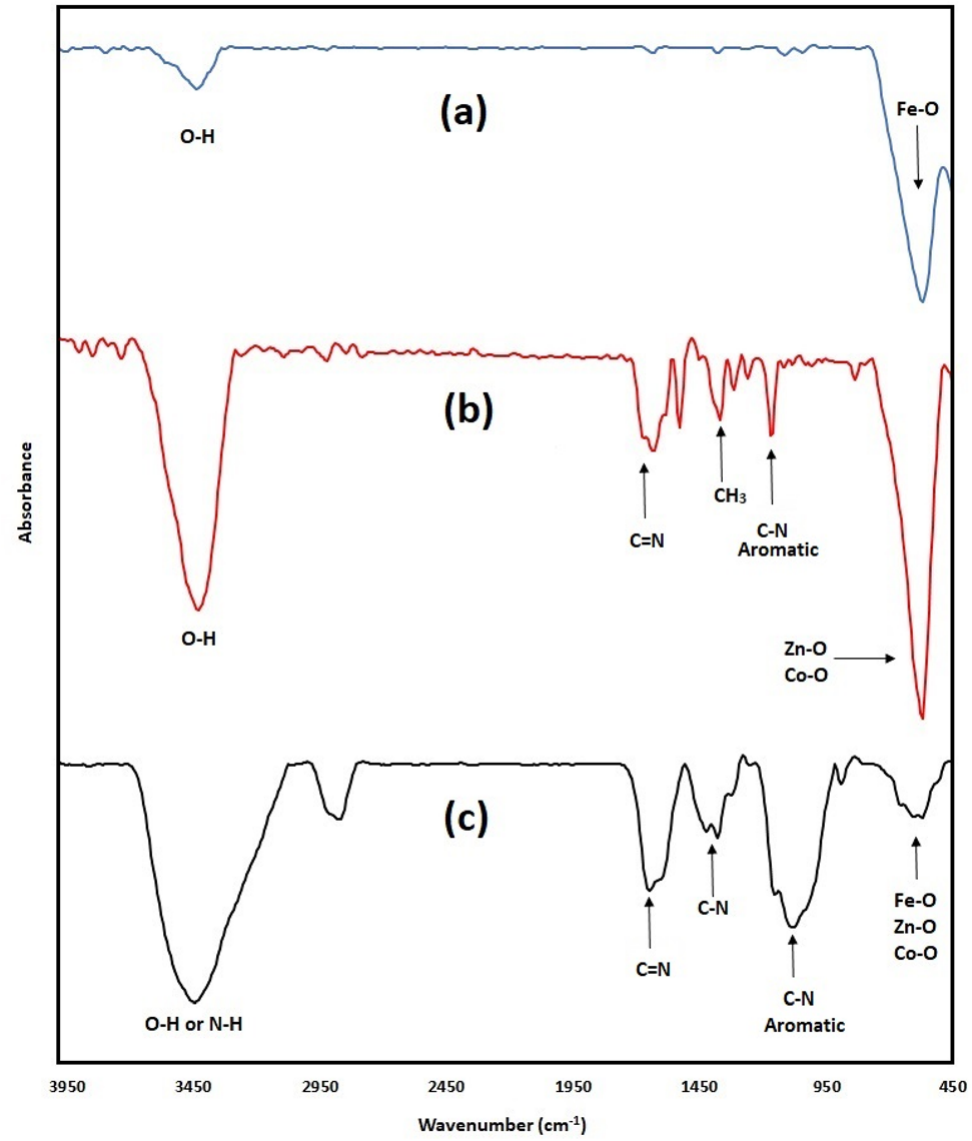
FTIR spectra of (a) Fe_3_O_4_ nanoparticles, (b) bimetallic Co/Zn ZIF-8, and (c) magnetic bimetallic Co/Zn ZIF-8@chitosan nanocomposite.

The FTIR spectra of Fe_3_O_4_ nanoparticles, bimetallic Co/Zn ZIF-8, and magnetic bimetallic Co/Zn ZIF-8@chitosan composite are presented in Fig. 3. The spectrum for Fe_3_O_4_ nanoparticles (Fig. 3a) displays a broad O–H stretching vibration at 3448 cm^−1^, attributable to adsorbed water. Crucially, the intense peak at 571 cm^−1^ corresponds to the Fe–O stretching vibration within the magnetite (Fe_3_O_4_) lattice, confirming its formation. The absence of peaks associated with organic functional groups (C–N, aromatic C–N) verifies its inorganic purity. The bimetallic Co/Zn ZIF-8 (Fig. 3b) exhibits a broad O–H stretch at 3440 cm^−1^, indicative of surface hydroxyls or moisture. Key peaks confirm the imidazolate linker of the ZIF-8 structure: the strong band at 1635 cm^−1^ is assigned to C=N stretching, while the peak at 1167 cm^−1^ is characteristic of aromatic C–N stretching within the imidazole ring. The definitive metal-linker coordination is evidenced by the Co–O or Zn–O stretching vibration band at 569 cm^−1^, signifying the bond between the Co^2+^/Zn^2+^ metal ions and the nitrogen atoms of the imidazolate anions. The magnetic bimetallic Co/Zn ZIF-8@chitosan composite (Fig. 3c) integrates features from all components. The broad peak at 3448 cm^−1^ arises from overlapping O–H stretching (chitosan, water, ZIF-8) and N–H stretching (chitosan amines). The C=N stretching vibration shifts to 1651 cm^−1^, potentially indicating modified electronic environments or hydrogen bonding within the composite. The emergence of a peak at 1421 cm^−1^ is attributed to C–N amine stretching from chitosan's primary amine groups. Peaks at 1080 cm^−1^, 894 cm^−1^, and 655 cm^−1^ correspond to various C–O stretching and deformation modes characteristic of chitosan's saccharide structure (C–O–C, C–OH). Most significantly, the distinct peak at 570 cm^−1^ demonstrates the presence of both the magnetic core and the ZIF framework, as it represents overlapping Fe–O stretching (from the Fe_3_O_4_ nanoparticles) and Co–O/Zn–O stretching (from the bimetallic ZIF-8).

### 2.5. Extraction procedure

A 15.0 mL aliquot of sample solution containing target aflatoxins (AFB_1_, AFB_2_, AFG_1_, AFG_2_) was transferred to a glass vial. Sodium chloride (0.375 g, 2.5% w/v) was added to enhance ionic strength, followed by pH adjustment to 7.4 using 1.0 mL of 0.1 M phosphate buffer (prepared from Na_2_HPO_4_/NaH_2_PO_4_). Magnetic sorbent (40.0 mg) was dispersed into the solution and agitated at 500 rpm for 8 min to facilitate adsorption. The sorbent was magnetically separated (NdFeB magnet, 1.4 T field strength; 2 min contact) and the supernatant decanted. For desorption, 120 µL HPLC-grade acetonitrile was introduced and vortex-mixed (500 rpm, 12.5 min). After magnetic separation (2 min), 20.0 µL of the acetonitrile phase was collected *via* microsyringe and injected into the HPLC-FLD system for quantitative analysis of aflatoxins.

### 2.6. Real sample preparation

Bottled commercial mineral waters from three companies, (Vata Brand, Pak Ab Sabalan Mineral Water Co, Ardabil, Iran), (Damavand Mineral Waters Co, Tehran, Iran), and (Miva Mineral Water Co, Amol, Iran), was purchased from a local supermarket (Mashhad, Iran). Agricultural samples included rice samples (*Oryza sativa* L. grains) of the traditional Hashemi cultivar and commercial Fajr brand obtained from retail suppliers in Mashhad, along with commercially available raw peanut kernels (*Arachis hypogaea* L.) procured from local supermarkets in the same metropolitan area. All samples were immediately transported to the laboratory under dark conditions at 4 °C, until analysis to preserve analyte integrity and prevent aflatoxin degradation.

Representative batches of peanut kernels or rice grains (10.0 g) are ground to achieve a homogeneous powder (particle size ≤0.5 mm), sieved through a 20-mesh screen (0.85 mm aperture), and dried at 60 °C for 24 h to standardize moisture content (<10% w/w). Aflatoxins are known to be thermally stable compounds, with studies confirming their stability during conventional drying processes at temperatures below 100 °C.^33,34^ Aliquots (0.2 ± 0.01 g) of dried matrix are extracted with acidified solvent (5.0 mL of acetonitrile: water: formic acid, 70 : 29 : 1 v/v/v) *via* vigorous shaking (250 rpm, 60 min). The mixture is centrifuged (5000×*g*, 10 min), and the supernatant is filtered through Whatman No. 4 filter paper. The filtrate is evaporated to dryness under N_2_ at 40 °C. The residue is reconstituted in 15.0 mL of deionized water and vortex-mixed (1 min) to yield a matrix-matched solution. For recovery studies, this solution is spiked with aflatoxin standards (AFB_1_/AFB_2_/AFG_1_/AFG_2_ in acetonitrile) to achieve final concentrations of 5.0 or 20.0 ng mL^−1^, followed by d-µSPE and HPLC-FLD analysis.

To prepare the bottled commercial mineral water samples, 50 mL of the water sample was selected and centrifuged at 5000 rpm for 10 minutes to remove suspended particulates. The supernatant is filtered through a 0.45 µm nylon membrane filter to eliminate residual colloids prior to dispersive micro solid-phase extraction (d-µSPE) and HPLC-FLD analysis. Then, the samples were spiked with a standard solution of Aflatoxin to achieve nominal concentrations of 5.0 or 20.0 ng mL^−1^. The spiked samples were reanalyzed to determine Aflatoxin concentration and calculate the recovery.

## 3 Results and discussions

### 3.1. The optimization of the procedure

#### 3.1.1. Type of sorbent

The optimization of sorbents for aflatoxin extraction was evaluated using four sorbents: Fe_3_O_4_, Zn ZIF-8, bimetallic Co/Zn ZIF-8, and bimetallic Co/Zn ZIF-8@chitosan. Initial extraction efficiencies (ER% ± standard deviation) revealed distinct performance differences (Table S1). All experiments were conducted in triplicate under identical experimental conditions, and the resulting data were used for both quantitative evaluation of sorbent performance and subsequent statistical analysis. To statistically validate these results, normality testing was first conducted *via* the Shapiro–Wilk test. All sorbents exhibited non-significant deviations from normality *p*-value > 0.05), confirming parametric test assumptions (Table S2). Subsequent one-way ANOVA demonstrated highly significant differences among the sorbents (*F*(3, 8) = 80.968, *p*-value < 0.001), with between-group variation (sum of squares = 2543.570) significantly exceeding within-group variation (sum of squares = 83.772) (Table S3). Post-hoc Tukey HSD tests further elucidated inter-sorbent differences (Table S4). Bimetallic Co/Zn ZIF-8@chitosan outperformed all others (*p*-value < 0.001), showing mean efficiency gains of 41.02% over Fe_3_O_4_, 20.44% over Zn ZIF-8, and 17.50% over bimetallic Co/Zn ZIF-8. The bimetallic Co/Zn ZIF-8 and Zn ZIF-8 both significantly surpassed Fe_3_O_4_ (*p*-value < 0.001), but no significant difference existed between them (*p*-value = 0.693). Similarly, while bimetallic Co/Zn ZIF-8@chitosan was superior to its non-chitosan counterpart (*p*-value = 0.001), the unmodified bimetallic Co/Zn ZIF-8 did not statistically exceed Zn ZIF-8 (*p*-value = 0.693). These results demonstrate that bimetallic Co/Zn ZIF-8@chitosan is the optimal sorbent for aflatoxin extraction, leveraging chitosan functionalization to significantly enhance efficiency. To provide a quantitative understanding of the adsorption behavior, the extraction efficiency data (Table S1) were correlated with the physicochemical properties of the sorbents characterized in Section 2.4. The adsorption capacity (*Q*, ng mg^−1^) for each sorbent was calculated based on the initial aflatoxin concentration (10 ng mL^−1^), sample volume (15 mL), and sorbent mass (40 mg), using the equation:
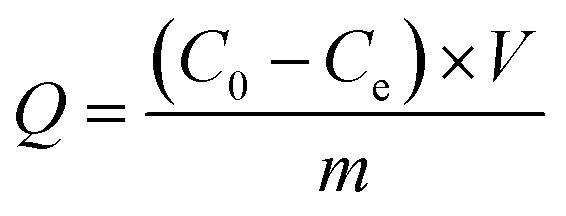
where *C*_0_ and *C*_e_ are the initial and equilibrium concentrations (ng mL^−1^), *V* is the sample volume (mL), and *m* is the sorbent mass (mg). The calculated adsorption capacities followed the order: Fe_3_O_4_ (1.44 ng mg^−1^) < Zn ZIF-8 (2.24 ng mg^−1^) < bimetallic Co/Zn ZIF-8 (2.38 ng mg^−1^) < bimetallic Co/Zn ZIF-8@chitosan (2.89 ng mg^−1^). The contribution of each sorbent component to the overall extraction was quantitatively assessed. The Fe_3_O_4_ core contributed approximately 1.44 ng mg^−1^ (38.45% extraction efficiency), attributable primarily to surface adsorption given its non-porous nature as confirmed by SEM (Section 2.4). The ZIF-8 framework enhanced the capacity by 0.80 ng mg^−1^ (55.6% increase over Fe_3_O_4_), corresponding to the high surface area and microporous structure that facilitates π–π stacking interactions with aflatoxin aromatic rings. The bimetallic Co/Zn ZIF-8 showed a further increase of 0.14 ng mg^−1^ (6.3% improvement over Zn ZIF-8), which can be attributed to the enhanced Lewis acidity of Co^2+^ sites. Based on the Co/Zn ratio (1 : 4) determined by EDX analysis (Section 2.4), the contribution of Co^2+^ sites to Lewis acid–base interactions with aflatoxin carbonyl groups was estimated at approximately 0.035 ng mg^−1^ per 1% Co incorporation. Most significantly, chitosan functionalization contributed an additional 0.51 ng mg^−1^ (21.4% improvement over bimetallic Co/Zn ZIF-8). This enhancement correlates with the density of amino and hydroxyl groups introduced by chitosan. Based on the chitosan content (38.5 wt% determined based on the proportions of components added in the sorbent synthesis step of Section 2.3.2), the contribution of chitosan functional groups was approximately 3.4 ng mg^−1^ per mg of chitosan, highlighting the efficiency of hydrogen bonding interactions at the optimal pH of 7.4.

The observed differences in aflatoxin extraction efficiency among the sorbents can be attributed to their distinct structural and functional properties, which directly influence their affinity for aflatoxins. Fe_3_O_4_ nanoparticles exhibit the lowest extraction efficiency (38.45% ± 1.80), primarily due to their lack of intrinsic porosity and limited functional groups for specific interactions with aflatoxins. In contrast, Zn ZIF-8 and bimetallic Co/Zn ZIF-8 leverage the inherent advantages of MOFs, including high surface area, tunable pore sizes, and hydrophobic cavities that facilitate π–π stacking interactions with the aromatic ring of aflatoxins. Beyond the statistical validation, the mechanistic basis for the enhanced performance of bimetallic Co/Zn ZIF-8 warrants closer examination. The partial substitution of Zn^2+^ with Co^2+^ within the ZIF-8 framework introduces two complementary effects that collectively improve aflatoxin affinity. First, Co^2+^ possesses higher Lewis acidity compared to Zn^2+^ due to its electronic configuration and unpaired d-electrons. This enhanced Lewis acidity strengthens acid–base interactions with the electron-rich carbonyl and lactone groups present in aflatoxin structures (*e.g.*, the β-dicarbonyl system of aflatoxin B_1_). Specifically, Co^2+^ sites can act as stronger electron pair acceptors, facilitating coordination-like interactions with the oxygen atoms of aflatoxin's lactone ring and furan moieties—functional groups critical to aflatoxin toxicity and planarity. Second, the incorporation of Co^2+^ subtly modifies the pore environment of ZIF-8. Although the overall SOD topology is preserved, the slightly different ionic radius and coordination preferences of Co^2+^*versus* Zn^2+^ can induce local distortions or changes in framework flexibility. This may create pore apertures with enhanced shape complementarity for the rigid, planar aflatoxin molecules, improving steric accessibility to internal adsorption sites. Additionally, the bimetallic nodes may generate heterogeneous surface charge distributions, promoting stronger electrostatic fields within the pores that polarize aflatoxin molecules and enhance physisorption *via* dipole–dipole and π-cation interactions. Importantly, while these bimetallic effects alone provided measurable improvement over monometallic ZIF-8, the modest (non-significant) statistical gain suggests that the intrinsic MOF architecture—while essential for high surface area and porosity—requires complementary functionalization to fully exploit multi-modal binding.

The substantial performance enhancement achieved by chitosan functionalization (17.50% increase over unmodified bimetallic Co/Zn ZIF-8; *p* = 0.001) necessitates detailed mechanistic consideration of chitosan's role in aflatoxin adsorption, particularly under the optimal extraction conditions (pH 7.4). Chitosan, a linear polysaccharide composed of randomly distributed β-(1 → 4)-linked d-glucosamine (deacetylated unit) and *N*-acetyl-d-glucosamine (acetylated unit), provides two key functional groups: primary amino (–NH_2_) and hydroxyl (–OH) groups. At the optimal extraction pH of 7.4, which is above chitosan's p*K*_a_ (∼6.5), the amino groups exist predominantly in their neutral deprotonated form (–NH_2_). Under these conditions, electrostatic interactions are minimal, and the primary binding mechanism shifts to hydrogen bonding. The neutral –NH_2_ groups act as effective hydrogen bond donors/acceptors, forming strong interactions with the oxygen-containing moieties of aflatoxins, particularly the lactone carbonyl and furan ring oxygen. Concurrently, the abundant –OH groups contribute additional hydrogen bonding sites, creating a dense network of polar interactions that effectively capture aflatoxin molecules. Additionally, chitosan's hydrophobic backbone segments may contribute to weak hydrophobic interactions with the aromatic rings of aflatoxins, complementing the π–π stacking provided by the MOF framework.

The cross-linking of chitosan with sodium tripolyphosphate (TPP) serves multiple critical functions beyond simple stabilization. TPP, a polyanion, cross-links chitosan chains through ionic gelation–electrostatic interactions between TPP's phosphate groups and chitosan's protonated amino groups during synthesis (typically conducted under acidic conditions). Once formed, this cross-linked structure remains stable at pH 7.4 and fundamentally transforms the chitosan architecture: (i) it converts soluble chitosan into an insoluble, mechanically stable hydrogel network that remains intact during extraction and desorption steps; (ii) it creates a three-dimensional porous structure with enhanced surface area and accessibility, as the cross-linked network prevents dense chain packing and maintains open channels for analyte diffusion; and (iii) it introduces additional phosphate groups that may participate in hydrogen bonding with aflatoxins at pH 7.4, further enriching the binding landscape.

Crucially, the cross-linked chitosan coating on the bimetallic MOF surface does not merely encapsulate the sorbent but creates a synergistic interface. The porous chitosan network allows aflatoxin molecules to diffuse through and access both the chitosan binding sites and, potentially, the underlying MOF surface. This dual-access architecture effectively increases the density of available binding sites while maintaining the high surface area advantages of the MOF core. Furthermore, the hydrophilic nature of the cross-linked chitosan enhances the sorbent's dispersibility in aqueous food matrices, improving mass transfer and extraction kinetics. Thus, the superior performance of bimetallic Co/Zn ZIF-8@chitosan arises from a hierarchical multi-modal binding mechanism optimized for pH 7.4: the MOF core provides high surface area and π–π stacking interactions with aflatoxin aromatic systems, while the cross-linked chitosan shell contributes abundant hydrogen bonding sites *via* its neutral –NH_2_ and –OH groups, with its porous architecture ensuring these sites remain accessible. This synergy between the bimetallic framework and the functional biopolymer coating explains the statistically significant enhancement observed.

#### 3.1.2. Type of desorption solvent

The optimization of desorption solvents for aflatoxin extraction was evaluated using five solvents: methanol, ethanol, 2-propanol, acetonitrile, and acetone. Extraction efficiencies (ER% ± standard deviation) revealed distinct performance variations (Table S5). Normality testing *via* the Shapiro–Wilk test confirmed all datasets adhered to a normal distribution (*p*-value > 0.05), satisfying parametric test assumptions (Table S6). One-way ANOVA demonstrated significant differences among solvents (*F*(4, 10) = 13.970, *p*-value < 0.001), with between-group variation (sum of squares = 495.188) substantially exceeding within-group variation (sum of squares = 88.614) (Table S7). Post-hoc Tukey HSD analysis delineated solvent-specific performance (Table S8). Acetonitrile exhibited significantly higher efficiency than ethanol (*p*-value = 0.027), 2-propanol (*p*-value = 0.024), and acetone (*p*-value < 0.001), though its advantage over methanol was marginally non-significant (*p*-value = 0.052). Methanol, ethanol, and 2-propanol showed no statistical differences (*p*-value > 0.986), forming a homogeneous subgroup. Acetone underperformed significantly *versus* all solvents (*p*-value < 0.027) except 2-propanol (*p*-value = 0.027), with the largest efficiency deficit observed against acetonitrile (18.11%, *p*-value < 0.001). These results highlight acetonitrile as the optimal desorption solvent, attributable to its intermediate polarity (dielectric constant *ε* = 37.5, 20 °C), aprotic nature, and strong dipole moment (3.2 D), which enhance solvation of moderately polar aflatoxins through dipole–dipole and van der Waals interactions. Methanol's comparable performance (*p*-value = 0.052) reflects its similar polarity (*ε* = 32.6, 20 °C) and protic character, enabling hydrogen bonding with aflatoxin lactone groups. Acetone's inferiority stems from its lower polarity (*ε* = 20.6, 20 °C) and potential ketone-aldehyde reactivity with aflatoxins, reducing extraction efficacy.^35^

#### 3.1.3. Screening step

Extraction efficiency in D-µ-SPE depends critically on multiple interacting factors, including pH, sorbent mass, and temporal parameters. Implementing a systematic experimental design is essential to screen these factors efficiently while minimizing resource-intensive trial-and-error approaches. The Definitive Screening Design (DSD) is particularly advantageous for this purpose, enabling robust evaluation of *k* factors with only 2 *k* + 1 experimental runs.^36,37^ This design simultaneously identifies dominant main effects, detects curvature, and reveals potential factor interactions, thereby accelerating optimization of the D-µ-SPE process.^38,39^ The approach enhances methodological robustness, reduces experimental costs, and expedites the development of reliable aflatoxin analysis protocols.

Seven continuous factors were investigated: sample solution volume (*A*), pH (*B*), sorbent mass (*C*), extraction time (*D*), desorption solvent volume (*E*), desorption time (*F*), and NaCl concentration (*G*). The DSD matrix (Table S9) details coded factor levels (−1, 0, +1) corresponding to experimentally defined minima, center points, and maxima. Analysis of variance (ANOVA) at 95% confidence (*α* = 0.05) was applied to extraction recovery (ER%) data (Table S10), with *p*-values < 0.05 indicating statistical significance. ER% values represent the mean of triplicate measurements performed under identical experimental conditions.

The ANOVA results demonstrate a statistically significant overall model (*p*-value = 0.0077), confirming that the factors collectively explain substantial variability in ER% (Table S10). Three factors significantly influenced aflatoxin extraction: pH (*B*) exhibited the strongest effect (*p*-value = 0.0042), underscoring its critical role in extraction efficiency. Extraction time (*D*) (*p*-value = 0.0106) and desorption solvent volume (*E*) (*p*-value = 0.0094) also showed significant impacts, highlighting their importance in the adsorption–desorption equilibrium. In contrast, desorption time (*F*) approached significance (*p*-value = 0.0556) but exceeded the 95% confidence threshold. The remaining factors—sample volume (*A*) (*p*-value = 0.9193), sorbent mass (*C*) (*p*-value = 0.4081), and NaCl concentration (*G*) (*p*-value = 0.2984)—were statistically insignificant, indicating minimal influence on ER% within the tested ranges.

These findings streamline process optimization by identifying pH, extraction time, and desorption solvent volume as key manipulable variables. Insignificant factors were fixed at their DSD center points: sample volume at 15.0 mL, sorbent mass at 40.0 mg, desorption time at 12.5 min, and NaCl concentration at 2.50% (w/v) during subsequent optimization phases.

#### 3.1.4. Optimization step

Central Composite Design (CCD) is a robust response surface methodology (RSM) tool widely employed for process optimization in analytical chemistry. It efficiently models complex nonlinear relationships by augmenting a factorial or fractional factorial design with axial (star) points and center replicates.^40^ This structure enables precise estimation of quadratic effects and interaction terms while maintaining rotatability—a critical feature ensuring consistent prediction accuracy across the experimental domain.^41^ CCD minimizes the experimental runs compared to full factorial designs, making it ideal for refining multifactor systems where curvature and interdependencies significantly influence outcomes.^42^ In this study, CCD was applied to optimize the three statistically significant factors identified for aflatoxin extraction—pH (*B*), extraction time (*D*), and desorption solvent volume (*E*)—from the definitive screening design results (Table 1). Insignificant factors (*A*, *C*, *F*, *G*) were fixed at their center-point values (15.0 mL, 40.0 mg, 12.5 min, 2.50% w/v, respectively). ER% was quantified as the arithmetic mean of three independent experimental repetitions conducted under consistent parameters.

Central composite design matrix for optimizing the significant factors

**Table:** 

Factor	Name	Units	Type	SubType	Minimum	Maximum	Mean	Std. Dev.
A	pH	—	Numeric	Continuous	5.00	8.00	6.50	1.09
B	Extraction time	min	Numeric	Continuous	5.00	10.00	7.50	1.81
C	Desorption solvent volume	µL	Numeric	Continuous	100.00	200.00	150.00	36.27

**Table:** 

Standard run	Run	A: pH	B: Extraction time	C: Desorption solvent volume	ER%
2	1	1	−1	−1	73.36
12	2	0	1	0	89.04
8	3	1	1	1	84.18
9	4	−1	0	0	76.05
18	5	0	0	0	93.56
13	6	0	0	−1	90.66
11	7	0	−1	0	73.32
3	8	−1	1	−1	73.46
16	9	0	0	0	93.87
6	10	1	−1	1	48.04
14	11	0	0	1	75.68
5	12	−1	−1	1	47.72
17	13	0	0	0	95.49
10	14	1	0	0	89.31
7	15	−1	1	1	58.86
19	16	0	0	0	96.07
15	17	0	0	0	90.68
4	18	1	1	−1	91.66
20	19	0	0	0	94.35
1	20	−1	−1	−1	68.16

The ANOVA results for the Central Composite Design (Table S11) demonstrate a statistically significant quadratic model (*p* < 0.0001) for aflatoxin extraction using magnetic bimetallic Co/Zn ZIF-8@chitosan, explaining 98.3% of the variability in extraction recovery (ER%). The model's high *F*-value (63.87) and non-significant lack of fit (*p* = 0.1105) confirm its robustness for optimization. All three linear factors exhibit strong significance: pH (*A*) (*p* < 0.0001, *F* = 51.06) dominates due to its control over sorbent-analyte electrostatic interactions; extraction time (*B*) (*p* < 0.0001, *F* = 98.66) underscores kinetic limitations in adsorption; and desorption solvent volume (*C*) (*p* < 0.0001, *F* = 90.23) dictates elution efficiency. Significant quadratic curvature (*p* ≤ 0.0011 for *A*^2^, *B*^2^, *C*^2^) reveals non-monotonic responses, confirming that efficiency maxima exist within the experimental domain. Interaction analysis identifies two significant binary effects: pH × extraction time (AB) (*p* = 0.0006) demonstrates synergy (*e.g.*, alkaline pH extends optimal binding duration), and extraction time × solvent volume (*BC*) (*p* = 0.0125) indicates time-dependent solvent efficacy for desorption. The non-significant pH × solvent volume (*AC*) interaction (*p* = 0.7798) implies these factors operate independently. The low residual error (SS = 76.02) relative to total variation further validates model adequacy. These results necessitate a balanced optimization strategy that accounts for the parabolic responses of individual factors and leverages the synergistic AB and BC interactions to maximize ER%.

**Fig. 4:**
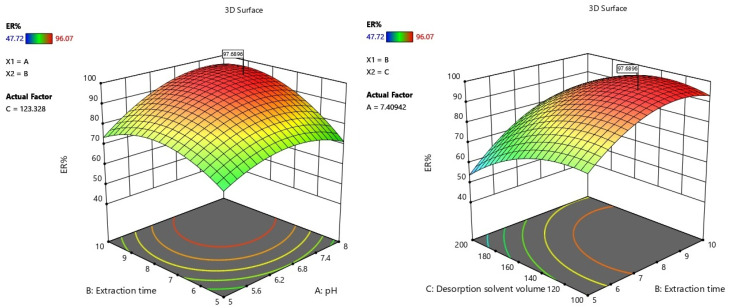
Response surface plots of significant binary interactions affecting aflatoxin extraction recovery (ER%).

The response surfaces in Fig. 4 illustrate how interactions between critical factors govern extraction efficiency, directly linked to the structural properties of magnetic bimetallic Co/Zn ZIF-8@chitosan and the chemical nature of aflatoxins. In plot (a), the synergistic interaction between extraction time (*B*) and desorption solvent volume (*C*) shows ER% peaking at intermediate values (120 µL solvent, 8 min). This occurs because aflatoxins (nonpolar polycyclic compounds with lactone and furan moieties) initially bind strongly to magnetic bimetallic Co/Zn ZIF-8@chitosan *via* hydrophobic interactions and π–π stacking within ZIF-8's porous framework. However, prolonged extraction saturates binding sites, while insufficient solvent volume (<120 µL) fails to disrupt these interactions during desorption due to chitosan's high affinity for mycotoxins. Optimal solvent volumes disrupt hydrogen bonding between aflatoxins and chitosan's hydroxyl/amino groups while solubilizing toxins.

Plot (b) reveals a pronounced interaction between pH (*A*) and extraction time (*B*), with maximum ER% at pH 7.4 and 8 min. At low pH (<6), protonation of chitosan's amino groups enhances electrostatic attraction to aflatoxins but destabilizes ZIF-8's Zn/Ni coordination bonds, reducing porosity. At alkaline pH (>8), deprotonated chitosan loses affinity, while aflatoxin lactone rings hydrolyze. The time-dependence arises because near-neutral pH balances: (1) ZIF-8's metal–organic framework stability for size-selective adsorption of aflatoxins, and (2) chitosan's hydrogen-bonding capacity, requiring sufficient time for toxin diffusion into bimetallic pores but avoiding sorbent degradation. These interactions confirm that extraction efficiency depends critically on harmonizing sorbent stability (ZIF-8) with dynamic binding mechanisms (chitosan), where mismatched pH/time or solvent/time conditions cause irreversible toxin trapping or sorbent collapse.

The quadratic model for aflatoxin extraction recovery is described by the equation:ER% = 92.67 + 6.23*A* + 8.66*B* − 8.28*C* + 4.75*AB* + 0.28*AC* + 2.96*BC* − 7.99*A*^2^ − 9.49*B*^2^ − 7.50*C*^2^,where *A* (pH), *B* (extraction time), and *C* (desorption solvent volume) are coded factors. The equation quantifies both directional and curvature effects: positive linear coefficients for pH (*A*) and extraction time (*B*) indicate enhanced recovery with increased values, while the negative coefficient for solvent volume (*C*) suggests reduced efficiency at higher volumes. Interaction terms reveal synergistic effects, particularly for AB (pH × extraction time) and BC (extraction time × desorption solvent volume). The significant negative quadratic coefficients (*A*^2^, *B*^2^, *C*^2^) confirm concave-downward curvature, indicating optimal recovery occurs at intermediate factor levels. Fit statistics (Table S12) validate the model's robustness: a high *R*^2^ (0.9829) indicates that 98.3% of ER% variability is explained by the model, closely aligned with the adjusted *R*^2^ (0.9675). The predicted *R*^2^ (0.8596) demonstrates reliable forecasting capability, while adequate precision (24.33) confirms a strong signal-to-noise ratio (>4 is desirable). Low standard deviation (2.76) and coefficient of variation (3.44%) reflect minimal experimental error and high reproducibility.

The optimum extraction conditions for maximizing aflatoxin recovery using magnetic bimetallic Co/Zn ZIF-8@chitosan were determined as pH 7.4, extraction time 8 min, and desorption solvent volume 120 µL. At these settings, the quadratic model predicted a mean ER% of 97.69%, with a 95% confidence interval (Table S13).

#### 3.1.5. Figure of merit

The analytical performance of the method for determining aflatoxins in water, rice, and peanut samples was evaluated through established figures of merit, as detailed in Table 2. Linearity, representing the concentration range over which the instrument response is proportional to the analyte concentration, was determined by analyzing serially diluted standard solutions. For water, linear ranges spanned 0.02–120 ng mL^−1^ across procedures, while rice (0.18–107 ng mL^−1^) and peanuts (0.19–105 ng mL^−1^) showed slightly narrower ranges due to matrix complexity. The coefficient of determination (*R*^2^), calculated *via* linear regression of calibration curves, exceeded 0.993 for all samples and procedures, confirming robust linearity. Limit of Detection (LOD), defined as the lowest concentration detectable at a signal-to-noise ratio of 3, was determined through blank sample analysis. Water exhibited the lowest LODs (0.006–0.009 ng mL^−1^), whereas rice and peanuts showed higher values (0.06–0.07 ng mL^−1^), reflecting matrix interference. Limit of Quantification (LOQ), representing the lowest reliably quantifiable concentration (signal-to-noise ratio of 10), followed a similar trend: water achieved 0.02–0.03 ng mL^−1^, while rice (0.18–0.21 ng mL^−1^) and peanuts (0.19–0.21 ng mL^−1^) required higher concentrations. Pre-concentration factors, calculated as the ratio of post-concentration to pre-concentration analyte levels, demonstrated effective enrichment: water (674–692), rice (652–674), and peanuts (649–682). Precision, expressed as Relative Standard Deviation (RSD), was assessed at low (0.4 ng mL^−1^) and high (40 ng mL^−1^) concentrations. Intra-day RSDs (repeatability; *n* = 3) ranged from 3.7–4.7% for all samples, indicating excellent reproducibility. Inter-day RSDs (intermediate precision; *n* = 3) were slightly higher (4.5–6.2%), attributable to day-to-day variability, but remained within acceptable limits.

**Table 2:** Analytical performance parameters for the determination of aflatoxins in water, rice, and peanut samples

Parameter	Sample		AFB1	AFB2	AFG1	AFG2
Linearity (ng mL^−1^)	Water		0.02–120	0.03–125	0.03–120	0.02–120
	Rice		0.18–98	0.20–105	0.18–107	0.23–101
	Peanuts		0.20–102	0.19–103	0.20–105	0.21–98
*R* ^2^	Water		0.9961	0.9947	0.9952	0.9938
	Rice		0.9945	0.9936	0.9945	0.9932
	Peanuts		0.9946	0.9932	0.9928	0.9919
LOD (ng mL-1)	Water		0.006	0.009	0.009	0.006
	Rice		0.05	0.06	0.05	0.07
	Peanuts		0.06	0.06	0.06	0.06
LOQ (ng mL-1)	Water		0.02	0.03	0.03	0.02
	Rice		0.18	0.20	0.18	0.21
	Peanuts		0.20	0.19	0.20	0.21
Pre-concentration factor	Water		683.2	674.7	692.1	679.7
	Rice		654.9	651.5	673.8	662.5
	Peanuts		649.3	659.2	682.4	659.7
RSD (*n* = 3) Intra-day	Water	0.4 ng mL^−1^	3.8	4.1	3.9	4.2
	Rice		4.4	4.7	4.3	4.7
	Peanuts		4.2	4.6	4.5	4.6
	Water	4.0 ng mL^−1^	3.8	3.9	3.8	3.9
	Rice		4.1	4.2	4.3	4.5
	Peanuts		4.3	4.2	4.1	4.7
	Water	40.0 ng mL^−1^	3.7	3.9	3.9	3.8
	Rice		4.1	4.3	4.1	4.2
	Peanuts		3.9	4.2	4.2	4.1
RSD (*n* = 3) Inter-day	Water	0.4 ng mL^−1^	5.6	5.6	5.7	5.7
	Rice		5.9	6.1	6.2	6.0
	Peanuts		6.0	6.2	5.9	5.9
	Water	4.0 ng mL^−1^	5.5	5.1	5.6	5.4
	Rice		5.8	5.4	5.9	5.7
	Peanuts		5.7	5.6	6.0	5.7
	Water	40.0 ng mL^−1^	5.3	5.1	5.2	4.5
	Rice		5.6	5.3	5.6	5.3
	Peanuts		5.7	5.7	5.6	5.4

### 3.2. Real sample analysis

The results from the recovery studies in spiked real samples demonstrate the accuracy and applicability of the dispersive micro-solid phase extraction/HPLC-FLD method for determining aflatoxins in complex matrices. For all water samples (Vata mineral water, Damavand mineral water, and Miva mineral water), no aflatoxins (AFB1, AFB2, AFG1, AFG2) were detected in unspiked samples (0.0 ng mL^−1^), confirming the absence of endogenous interferences. At the 5.0 ng mL^−1^ spike level, mean recoveries ranged from 93.0% (AFG1 in Miva mineral water) to 95.2% (AFG1 in Damavand mineral water), with precision (RSD) values between 3.2–4.3% (reflected by ± SD values of 0.15–0.20 ng mL^−1^). At the higher 20.0 ng mL^−1^ spike, recoveries improved to 97.1–98.0% across all mineral water types, with RSDs of 3.9–4.2% (±SD 0.78–0.82 ng mL^−1^), indicating excellent method robustness in aqueous environments (Table 3).

**Table 3:** Determination of Aflatoxins in spiked real water, rice, and peanut samples (*n* = 3)

Sample	Spike(ng mL^−1^)		AFB1	AFB2	AFG1	AFG2
Mineral water^a^	0.0	Found (ng mL^−1^)	ND^b^	ND	ND	ND
		Recovery (%)	—	—	—	—
	5.0	Found (ng mL^−1^)	4.71 ± 0.16	4.72 ± 0.17	4.71 ± 0.17	4.70 ± 0.15
		Recovery (%)	94.2	94.4	94.2	94.0
	20.0	Found (ng mL^−1^)	19.55 ± 0.80	19.56 ± 0.81	19.60 ± 0.79	19.58 ± 0.81
		Recovery (%)	97.8	97.8	98.0	97.9
Mineral water^c^	0.0	Found (ng mL^−1^)	ND	ND	ND	ND
		Recovery (%)	—	—	—	—
	5.0	Found (ng mL^−1^)	4.73 ± 0.18	4.70 ± 0.19	4.76 ± 0.20	4.73 ± 0.17
		Recovery (%)	94.6	94.0	95.2	94.6
	20.0	Found (ng mL^−1^)	19.48 ± 0.79	19.50 ± 0.81	19.46 ± 0.78	19.45 ± 0.80
		Recovery (%)	97.4	97.5	97.3	97.2
Mineral water^d^	0.0	Found (ng mL^−1^)	ND	ND	ND	ND
		Recovery (%)	—	—	—	—
	5.0	Found (ng mL^−1^)	4.70 ± 0.19	4.67 ± 0.19	4.65 ± 0.18	4.68 ± 0.20
		Recovery (%)	94.0	93.4	93.0	93.6
	20.0	Found (ng mL^−1^)	19.51 ± 0.81	19.46 ± 0.79	19.47 ± 0.81	19.42 ± 0.82
		Recovery (%)	97.6	97.3	97.4	97.1
Rice^e^	0.0	Found (ng mL^−1^)	ND	ND	ND	ND
		Recovery (%)	—	—	—	—
	5.0	Found (ng mL^−1^)	4.56 ± 0.20	4.64 ± 0.19	4.63 ± 0.20	4.58 ± 0.20
		Recovery (%)	91.2	92.8	92.6	91.6
	20.0	Found (ng mL^−1^)	19.41 ± 0.82	19.43 ± 0.80	19.42 ± 0.81	19.45 ± 0.81
		Recovery (%)	97.0	97.2	97.1	97.2
Rice^f^	0.0	Found (ng mL^−1^)	ND	ND	ND	ND
		Recovery (%)	—	—	—	—
	5.0	Found (ng mL^−1^)	4.63 ± 0.21	4.61 ± 0.21	4.64 ± 0.19	4.65 ± 0.20
		Recovery (%)	92.6	92.2	92.8	93.0
	20.0	Found (ng mL^−1^)	19.51 ± 0.82	19.48 ± 0.83	19.45 ± 0.81	19.42 ± 0.80
		Recovery (%)	97.6	97.4	97.2	97.1
Peanuts	0.0	Found (ng mL^−1^)	0.61 ± 0.03	0.38 ± 0.03	0.29 ± 0.04	0.31 ± 0.04
		Recovery (%)	—	—	—	—
	5.0	Found (ng mL^−1^)	5.27 ± 0.20	5.03 ± 0.21	4.93 ± 0.20	4.95 ± 0.22
		Recovery (%)	93.2	93.0	92.8	92.8
	20.0	Found (ng mL^−1^)	19.47 ± 0.82	19.14 ± 0.83	18.94 ± 0.81	18.93 ± 0.83
		Recovery (%)	94.3	93.8	93.2	93.1

a Vata mineral water.

b Not detect.

c Damavand mineral water.

d Miva mineral water.

e Hashami rice.

f Fajr rice.

For Hashami rice and Fajr rice, unspiked samples also showed no detectable aflatoxins. Recoveries at 5.0 ng mL^−1^ were slightly lower (91.2–93.0% for rice^2^; 92.2–93.0% for rice^3^) but remained within acceptable validation limits, accompanied by RSDs of 4.0–4.4% (±SD 0.19–0.21 ng mL^−1^). At 20.0 ng mL^−1^, recoveries improved to 97.0–97.6% (RSDs: 4.0–4.3%; ±SD 0.80–0.83 ng mL^−1^), underscoring consistent accuracy despite the complex carbohydrate matrix. The satisfactory recovery rates obtained for spiked rice (91.2–97.6%) empirically confirm that any potential analyte loss during the drying step, if present, is negligible and does not affect the accuracy of the method.

Notably, unspiked peanuts contained low endogenous levels of aflatoxins (AFB_1_: 0.61 ± 0.03 ng mL^−1^; AFB_2_: 0.38 ± 0.03 ng mL^−1^; AFG_1_: 0.29 ± 0.04 ng mL^−1^; AFG_2_: 0.31 ± 0.04 ng mL^−1^). After spiking, recoveries at 5.0 ng mL^−1^ were 92.8–93.2% (RSDs: 3.8–4.4%; ±SD 0.20–0.22 ng mL^−1^), while at 20.0 ng mL^−1^, recoveries ranged from 93.1–94.3% (RSDs: 4.2–4.4%; ±SD 0.81–0.83 ng mL^−1^) (Table 3). These results confirm the method's efficacy in lipid-rich matrices despite pre-existing contamination. The consistent recoveries across both low (5.0 ng mL^−1^) and high (20.0 ng mL^−1^) spike levels further support the absence of thermal degradation under the selected drying conditions.

Collectively, the recovery rates (91.2–98.0%) and precision (RSDs ≤ 4.4%) across all samples and spike levels meet international validation criteria, affirming the method's reliability for quantifying trace aflatoxins in diverse environmental and agricultural samples.

An important consideration for any analytical method intended for food safety monitoring is its ability to detect aflatoxin concentrations below the maximum residue limits (MRLs) established by regulatory authorities. The European Union has set the maximum permissible levels for total aflatoxins (sum of AFB_1_, AFB_2_, AFG_1_, and AFG_2_) at 4.0 µg kg^−1^ (equivalent to 4.0 ng mL^−1^ for liquid matrices and 4.0 ng g^−1^ for solid matrices) in groundnuts and cereals intended for direct human consumption, with a specific limit of 2.0 µg kg^−1^ for AFB_1_ alone.^43^ The U.S. Food and Drug Administration (FDA) establishes action levels for total aflatoxins at 20 µg kg^−1^ in all food commodities, while the Codex Alimentarius Commission sets limits ranging from 0.5 to 15 µg kg^−1^ depending on the commodity.^44,45^ The Iran National Standard similarly sets limits of 5 µg kg^−1^ for AFB_1_ and 15 µg kg^−1^ for total aflatoxins in rice and peanuts.

As presented in Table 2, the LODs achieved by the proposed method range from 0.006–0.009 ng mL^−1^ in water, 0.05–0.07 ng mL^−1^ in rice, and 0.06 ng mL^−1^ in peanuts. These values are substantially lower than the most stringent international regulatory limits (*e.g.*, 2.0 ng g^−1^ for AFB_1_ in cereals under EU regulations). The LOQs (0.02–0.03 ng mL^−1^ for water; 0.18–0.21 ng mL^−1^ for rice and peanuts) also fall well below these thresholds, ensuring reliable quantification at legally relevant concentrations.

The practical significance of this sensitivity is demonstrated in the peanut samples, where naturally occurring aflatoxins were detected at concentrations of 0.29–0.61 ng mL^−1^ (Table 3). These endogenous levels, while detectable, remain below the EU MRL of 4.0 ng g^−1^ for total aflatoxins in peanuts, confirming that the sampled products complied with international safety standards. Furthermore, the spike recovery experiments at 5.0 and 20.0 ng mL^−1^ encompass concentrations both below and above regulatory limits, validating method performance across the entire range of interest for food safety monitoring.

### 3.3. Matrix effect evaluation

The matrix effect (ME) is a critical parameter in quantitative analysis, as co-extracted matrix components may enhance or suppress the analytical signal, potentially compromising accuracy. In this study, the matrix effect was evaluated by comparing the peak areas of aflatoxins spiked into blank matrix extracts after the extraction procedure (post-extraction spiking) with those of standard solutions prepared in neat solvent at the same concentration levels. The matrix effect percentage (% ME) was calculated using the following equation:
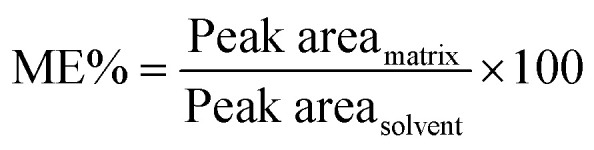


As shown in Table S14, the matrix effect for water samples was minimal, with % ME values ranging from 96.8% to 97.3% across all four aflatoxins. This negligible suppression (less than 4%) confirms that the simple aqueous matrix does not significantly interfere with analyte detection, consistent with the high recoveries observed in spiked water samples (94.0–98.0%). For rice samples, moderate signal suppression was observed, with % ME values ranging from 89.8% to 90.7%. This suppression (approximately 9–10%) is attributed to co-extracted carbohydrates and other matrix components that partially interfere with chromatographic behavior. The values remain within acceptable limits for complex food matrices and are effectively compensated by the matrix-matched calibration employed for rice analysis, as reflected in the satisfactory recovery rates (91.2–97.6%). Peanut samples exhibited the most pronounced matrix effect, with % ME values ranging from 86.1% to 87.5%. The signal suppression (approximately 12–14%) is attributable to the complex lipid-rich matrix of peanuts, which introduces co-extracted fatty acids and other hydrophobic compounds during sample preparation. The slightly higher suppression observed for AFB_2_ (86.1%) compared to AFB_1_ (87.3%) suggests minor differences in susceptibility to matrix interference based on analyte polarity. The varying degrees of matrix effect across the three matrices highlight the importance of matrix-specific method validation. The use of matrix-matched calibration standards for rice and peanut samples effectively compensates for the observed suppression, ensuring reliable quantification as confirmed by the satisfactory recovery rates (92.8–94.3% for peanuts).

### 3.4. Reusability and stability study of the sorbent

The reusability of the bimetallic Co/Zn ZIF-8@chitosan sorbent is a critical parameter for assessing its practical applicability and cost-effectiveness. Therefore, the reusability of the bimetallic Co/Zn ZIF-8@chitosan sorbent was evaluated by performing successive extraction–desorption cycles under optimized conditions. For each cycle, 40.0 mg of the magnetic sorbent was dispersed in 15.0 mL of sample solution containing aflatoxins at a concentration of 10 ng mL^−1^. The extraction procedure was carried out as described in Section 2.5, including pH adjustment to 7.4 with phosphate buffer, agitation at 500 rpm for 8 min, magnetic separation, and desorption with 120 µL acetonitrile (500 rpm, 12.5 min). After each cycle, the sorbent was washed twice with 1.0 mL acetonitrile followed by 1.0 mL ultrapure water to remove any residual analytes or matrix components, then conditioned with phosphate buffer (pH 7.4) prior to the next extraction cycle. The extraction efficiency (ER%) was calculated for each aflatoxin (AFB_1_, AFB_2_, AFG_1_, AFG_2_) in triplicate across five consecutive cycles. The extraction efficiency (ER%) for all four aflatoxins was monitored over five successive extraction–desorption cycles (Table S15). The sorbent exhibited excellent initial extraction efficiency exceeding 97% for all aflatoxins in the first cycle. A gradual decrease in efficiency was observed over subsequent cycles, with ER% remaining above 91% through four cycles and above 87% after five cycles. The standard deviations increased moderately with cycle number, reflecting slight variability introduced upon repeated use. The retention of high extraction efficiency (>87%) after five cycles demonstrates the sorbent's robust reusability and operational stability under the optimized extraction conditions (pH 7.4). The slight decline in extraction performance can be attributed to progressive saturation of active binding sites and minor physical changes in the sorbent structure during repeated acetonitrile desorption and washing steps. However, the cross-linked chitosan network plays a crucial protective role: the TPP-cross-linked hydrogel maintains structural integrity, preventing dissolution or significant swelling during acetonitrile exposure, while the magnetic Fe_3_O_4_ core retains its responsiveness throughout the cycles, enabling consistent magnetic separation. These results establish that the bimetallic Co/Zn ZIF-8@chitosan sorbent can be reused for at least four cycles with ER% > 91% and up to five cycles with acceptable performance, demonstrating its cost-effectiveness and suitability for routine analytical applications. For optimal reliability, replacement of the sorbent after four consecutive extractions is recommended.

### 3.5. Comparison with other methods

Table 4 compares the performance of the proposed dispersive micro-solid phase extraction (D-µ-SPE) method with various established and recently reported microextraction techniques for analyzing aflatoxins (AFB_1_, AFB_2_, AFG_1_, AFG_2_) in different food matrices. Overall, the D-µ-SPE method demonstrates significant advantages in sensitivity, linear range, precision, and recovery compared to most alternatives, while exhibiting some limitations in breadth of sample application testing. The D-µ-SPE method achieves remarkably low Limits of Detection (LOD), ranging from 0.006 to 0.009 ng mL^−1^ across all four aflatoxins. This sensitivity surpasses nearly all listed methods; for instance, AA-DLLME reports LODs of 0.13–0.68 ng mL^−1^,^46^ Modified-DLLME shows much higher LODs (10.0–15.0 ng mL^−1^),^47^ and while methods like NF–PSHF achieve a lower LOD for AFG_1_ (0.007 ng mL^−1^),^48^ D-µ-SPE maintains consistently ultra-low LODs for all analytes. When compared with recent bimetallic MOF-based methods, our LODs are either superior or comparable: Ghorbani *et al.*^11^ reported LODs of 0.01–0.02 ng mL^−1^ using magnetic chitosan-bimetallic MOF, while another D-µ-SPE method^49^ achieved 0.01–0.04 ng mL^−1^. Notably, a magnetic solid-phase extraction method^50^ reported an LOD of 0.02 µg kg^−1^ for AFB_1_ alone, though this method focused solely on AFB_1_ rather than simultaneous determination of all four aflatoxins. Furthermore, D-µ-SPE offers an exceptionally wide Linear Range (LR) of 0.02–125 ng mL^−1^, covering nearly four orders of magnitude. This range is substantially broader than most methods (*e.g.*, AA-DLLME: 0.08–10 ng mL^−1^,^46^ DLLME for vegetable milk: 0.24–10 ng mL^−1^,^51^ DLLME for tomato paste: 0.6–20 ng mL^−1^,^52^ NF–PSHF: 0.1–40 ng mL^−1^ (ref. 48)), providing greater analytical flexibility across concentration levels. Compared to recent bimetallic MOF methods, our linear range is wider than those reported by Ghorbani *et al.*^11^ (0.05–89.5 ng mL^−1^) and the D-µ-SPE method reported in ref. 49 (0.04–79.2 ng mL^−1^). The precision of D-µ-SPE, indicated by Relative Standard Deviation (RSD%) values between 3.7% and 4.7%, is excellent and highly consistent for all aflatoxins, outperforming methods with higher or more variable RSDs like AA-DLLME (≤14.2%)^46^ and rGO-PVPP-Reinforced HF (≤3.6%).^53^ This precision is comparable to recent bimetallic MOF methods: Ghorbani *et al.*^11^ reported RSDs of 3.7–4.7%, while the method in ref. 49 showed RSDs of 4.1–7.6%. Recovery rates for D-µ-SPE (91.2–97.9%) are consistently high, accurate, and show minimal variation (±∼5%), contrasting favorably with methods exhibiting lower or more erratic recoveries, such as rGO-PVPP-REINFORCED HF (60.2–108.2%)^53^ and FPSE (80.0–95.0%).^54^ The recoveries are comparable to those achieved by recent bimetallic MOF methods: 87.8–97.9% for Ghorbani *et al.*,^11^ 92.0–97.8% for the D-µ-SPE method in ref. 49, and 89.7–99.5% for MSPE in ref. 50. The method is validated across diverse samples (mineral water, rice, and peanuts), demonstrating its robustness. The primary strength of the D-µ-SPE method lies in its exceptional combination of ultra-high sensitivity (low LODs), a very wide working range, excellent precision, and high, consistent accuracy (recovery) for all four major aflatoxins simultaneously. This balanced performance profile is unmatched by any single method in Table 4. The wide linear range is particularly advantageous for analyzing samples with unpredictable or highly variable contamination levels. The main relative weakness is the narrower range of sample matrices explicitly listed in its validation (water, rice, peanuts) compared to some methods tested on broader categories like “Foods”^48,54^ or specific items like sesame, wheat, vegetable milk, or corn.^11,51,53^

**Table 4:** Comparison of the procedure with other microextraction methods

Analyte	Microextraction	Detection	Sample	LOD (ng mL^−1^)	LR (ng mL^−1^)	RSD %	Recovery	Ref.
AFB1	AA-DLLME^a^	HPLC-FLD	Rice	0.68	0.4–10	≤12.5	≥76.0	46
AFB2				0.13	0.08–2	≤11.6	≥81.3	
AFG1				0.68	0.4–10	≤14.2	≥88.9	
AFG2				0.13	0.08–2	≤10.5	≥89.3	
AFB1	DLLME	HPLC-FLD	Vegetable-based milk	—	0.32–10	0.6–6.2	88–104	51
AFB2				—	0.24–10	1.0–6.6	85–100	
AFG1				—	0.48–10	0.2–7.4	91–103	
AFG2				—	0.28–10	0.9–5.1	84–101	
AFB1	Modified-DLLME	HPLC-FLD	Grain	15.0	50–500^b^	5.1–6.4	97.7–102.6	47
AFB2				10.0	35–400^b^	4.7–6.0	97.6–101.2	
AFB1	rGO–PVPP-reinforced HF^c^	HPLC-FLD	Sesame	0.33^d^	—	2.6	71.8–108.2	53
AFB2			Wheat	0 × 10^d^	—	3.0	79.7–97.2	
AFG1			Rice	0.37^d^	—	3.6	60.2–77.95	
AFG2				0.1^d^	—	3.1	77.2–95.4	
AFB1	DLLME	HPLC-FLD	Tomato paste	0.14	0.6–20	—	91–94	52
AFB1	NF-PSHE^e^	HPLC-FLD	Foods	0.05	0.1–40	1.8–3.6	90.8–112.7	48
AFB1	PFSPE^f^	HPLC-FLD	Milk	0.13	0.2–20	2.81–6.43	80.22–96.21	55
AFB2				0.007	0.1–10			
AFG1				0.16	0.4–40			
AFG2				0.13	0.2–20			
AFB1	DµSPE	HPLC-FLD	Real water	0.01	0.05–82.6	3.9–4.6	88.4–97.6	11
AFB2			Herbal distillate	0.02	0.07–86.4	4.0–4.5	87.8–97.7	
AFG1			Black tea	0.02	0.08–85.7	3.7–4.4	87.8–97.9	
AFG2			Corn	0.02	0.07–89.5	4.2–4.7	88.2–97.6	
AFB1	FPSE^g^	HPLC-FLD	Foods	0.12–0.51	—	3.9–5.3	80.0–95.0	54
AFB2								
AFG1								
AFG2								
AFB1	DµSPE	HPLC-FLD	Real water	0.04	0.15–77.6	4.1–7.0	92.5–96.8	49
AFB2			Rice	0.01	0.04–64.8	4.1–6.4	93.0–97.7	
AFG1				0.04	0.15–79.2	4.1–7.6	92.0–97.5	
AFG2				0.03	0.11–69.5	4.1–7.0	92.5–97.8	
AFB1	MSPE	HPLC	Peanut, corn, rice, and wheat samples	0.02 µg Kg^−1^	—	—	89.7–99.5	50
AFB1	DµSPE	HPLC-FLD	Mineral water rice, and peanut samples	0.006	0.02–120	3.7–4.4	91.2–97.0	This work
AFB2				0.009	0.03–125	3.9–4.7	92.2–97.8	
AFG1				0.009	0.03–120	3.8–4.5	92.6–97.4	
AFG2				0.006	0.02–120	3.8–4.2	91.6–97.9	

a Air-assisted dispersive liquid–liquid microextraction.

b ng Kg^−1^.

c Reduced graphene oxide-polyvinyl poly pyrrolidone Reinforced hollow fiber.

d ng g^−1^.

e Nanofiber-packed solid-phase extraction.

f Packed-nanofiber solid-phase extraction.

g Fabric phase sorptive extraction, Magnetic solid-phase extraction.

## 4 Conclusion

This study successfully developed a highly sensitive and reliable D-µ-SPE method coupled with HPLC-FLD for the determination of carcinogenic aflatoxins. The integration of bimetallic Co/Zn ZIF-8 with chitosan and Fe_3_O_4_ nanoparticles yielded a novel magnetic composite sorbent, which demonstrated significantly enhanced extraction efficiency over monometallic ZIF-8. A sequential optimization strategy was implemented: Definitive Screening Design (DSD) first identified pH, extraction time, and desorption solvent volume as critical manipulable variables, while fixing insignificant factors at their center points (sample volume: 15.0 mL; sorbent mass: 40.0 mg; desorption time: 12.5 min; NaCl: 2.50% w/v). Subsequent Central Composite Design (CCD) optimization established ideal conditions (pH 7.4, extraction time 8 min, desorption solvent volume 120 µL), achieving a predicted mean extraction recovery of 97.69% (95% CI). The validated method exhibits exceptional sensitivity, wide linearity, and high precision. Crucially, its applicability was confirmed across critical matrices—mineral water (environmental contamination risks), rice (global dietary staple), and peanuts (highly susceptible crop)—yielding relative recoveries of 91.2–98.0% (RSD < 4.4%). This work advances both sorbent design for mycotoxin analysis and optimization methodologies, delivering a robust tool for food/environmental safety monitoring of high-risk commodities.

## Ethical statement

This article lacks research involving human or animal subjects.

## Author contributions

Foroughalzaman Kazempoor Mofrad: data curation, formal analysis, validation, writing – original draft. Alireza Shams: conceptualization, investigation, validation, writing e original draft, resources. Mahdi Ghorbani: methodology, formal analysis, investigation, validation, writing – review & editing.

## Conflicts of interest

The authors declare no competing interests.

## Supplementary Material

RA-016-D5RA09926A-s001

## Data Availability

All data generated or analyzed during this study are included in this published article and its supplementary information (SI) files. Supplementary information is available. See DOI: https://doi.org/10.1039/d5ra09926a.
