# Supplementary material for: Ultra-sensitive determination of carcinogenic aflatoxins in food matrices using a novel magnetic bimetallic ZIF-8@chitosan sorbent and dispersive micro-solid phase extraction
Source: RSC Adv. 2026 May 26;16(26):23528–43. doi: 10.1039/d5ra09926a (PMC13213553; doi:10.1039/d5ra09926a)
Supplement: RA-016-D5RA09926A-s001 [file RA-016-D5RA09926A-s001.pdf]

Supplementary Information (SI) for RSC Advances.
This journal is © The Royal Society of Chemistry 2026

Electronic Supplementary Materials

Ultra-Sensitive Determination of Carcinogenic Aflatoxins in Food Matrices Using a Novel Magnetic Bimetallic ZIF-8@Chitosan Sorbent and Dispersive Micro-Solid Phase Extraction

Alireza Shams[1*], Foroughalzaman Kazempoor Mofrad[1], Mahdi Ghorbani[2*]

[1] Department of Chemistry, Ma. C., Islamic Azad University, Mashhad, Iran

[2] Department of Chemistry, Faculty of Sciences, Ferdowsi University of Mashhad, Mashhad, Iran

*** Corresponding authors.**

Alireza Shams

E-mail address: Farsh1358@gmail.com

Mahdi Ghorbani

E-mail address: ghorbani267@yahoo.com, ghorbani267@mail.um.ac.ir

Table S1. Effect of the sorbent type on the Aflatoxin extraction

Sorbent	Fe_3O_4	Zn ZIF-8	Bimetallic Co/Zn ZIF-8	Bimetallic Co/Zn ZIF-8@ chitosan
ER%±S	38.45±1.80	59.02±2.16	61.96±1.94	79.46±1.52

Table S2. Test of normality for analyzing the sorbent type results on the Aflatoxin extraction

Sorbent	Kolmogorov-Smirnov[a]			Shapiro-Wilk		
	Statistic	df	Sig.	Statistic	df	Sig.
Fe3O4	.191	3	.	.997	3	.901
Zn ZIF-8	.214	3	.	.989	3	.802
Bimetallic Co/Zn ZIF-8	.175	3	.	1.000	3	.990
Bimetallic Co/Zn ZIF-8@chitosan	.204	3	.	.993	3	.845

a. Lilliefors Significance Correction

Table S3. ANOVA for analyzing the sorbent type results on the Aflatoxin extraction

	Sum of Squares	df	Mean Square	F	Sig.
Between Groups	2543.570	3	847.857	80.968	.000
Within Groups	83.772	8	10.472		
Total	2627.343	11			

Table S4. Tukey HSD for analyzing the sorbent type results on the Aflatoxin extraction

(I) Sorbent	(J) Sorbent	Mean Difference (I-J)	Std. Error	Sig.	95% Confidence Interval	
					Lower Bound	Upper Bound
Fe3O4	Zn ZIF-8	-20.57667*	2.64216	.000	-29.0378	-12.1155
	Bimetallic Co/Zn ZIF-8	-23.51333*	2.64216	.000	-31.9745	-15.0522
	Bimetallic Co/Zn ZIF-8@chitosan	-41.01667*	2.64216	.000	-49.4778	-32.5555
Zn ZIF-8	Fe3O4	20.57667*	2.64216	.000	12.1155	29.0378
	Bimetallic Co/Zn ZIF-8	-2.93667	2.64216	.693	-11.3978	5.5245
	Bimetallic Co/Zn ZIF-8@chitosan	-20.44000*	2.64216	.000	-28.9011	-11.9789
Bimetallic Co/Zn ZIF-8	Fe3O4	23.51333*	2.64216	.000	15.0522	31.9745
	Zn ZIF-8	2.93667	2.64216	.693	-5.5245	11.3978
	Bimetallic Co/Zn ZIF-8@chitosan	-17.50333*	2.64216	.001	-25.9645	-9.0422
Bimetallic Co/Zn ZIF-8@chitosan	Fe3O4	41.01667*	2.64216	.000	32.5555	49.4778
	Zn ZIF-8	20.44000*	2.64216	.000	11.9789	28.9011
	Bimetallic Co/Zn ZIF-8	17.50333*	2.64216	.001	9.0422	25.9645

*. The mean difference is significant at the 0.05 level.

Table S5. Effect of the desorption solvent type on the Aflatoxin extraction

Solvent	Methanol	Ethanol	2-Propanol	Acetonitrile	Acetone
ER%±S	79.46±1.52	78.42±1.63	78.27±1.95	87.40±1.21	69.29±2.13

Table S6. Test of normality for analyzing the desorption solvent type results on the Aflatoxin extraction

Desorption solvent	Kolmogorov-Smirnov[a]			Shapiro-Wilk		
	Statistic	df	Sig.	Statistic	df	Sig.
Methanol	.204	3	.	.993	3	.845
Ethanol	.205	3	.	.993	3	.839
1-propanol	.322	3	.	.880	3	.324
Acetonitrile	.195	3	.	.996	3	.882
Acetone	.191	3	.	.997	3	.900

Table S7. ANOVA for analyzing the desorption solvent type results on the Aflatoxin extraction

	Sum of Squares	df	Mean Square	F	Sig.
Between Groups	495.188	4	123.797	13.970	.000
Within Groups	88.614	10	8.861		
Total	583.803	14			

Table S8. Tukey HSD for analyzing the desorption solvent type results on the Aflatoxin extraction

(I) Desorption solvent	(J) Desorption solvent	Mean Difference (I-J)	Std. Error	Sig.	95% Confidence Interval	
					Lower Bound	Upper Bound
Methanol	Ethanol	1.04667	2.43056	.992	-6.9525	9.0458
	1-propanol	1.19667	2.43056	.986	-6.8025	9.1958
	Acetonitrile	-7.93667	2.43056	.052	-15.9358	.0625
	Acetone	10.17667*	2.43056	.013	2.1775	18.1758
Ethanol	Methanol	-1.04667	2.43056	.992	-9.0458	6.9525
	1-propanol	.15000	2.43056	1.000	-7.8492	8.1492
	Acetonitrile	-8.98333*	2.43056	.027	-16.9825	-.9842
	Acetone	9.13000*	2.43056	.024	1.1308	17.1292
1-propanol	Methanol	-1.19667	2.43056	.986	-9.1958	6.8025
	Ethanol	-.15000	2.43056	1.000	-8.1492	7.8492
	Acetonitrile	-9.13333*	2.43056	.024	-17.1325	-1.1342
	Acetone	8.98000*	2.43056	.027	.9808	16.9792
Acetonitrile	Methanol	7.93667	2.43056	.052	-.0625	15.9358
	Ethanol	8.98333*	2.43056	.027	.9842	16.9825
	1-propanol	9.13333*	2.43056	.024	1.1342	17.1325
	Acetone	18.11333*	2.43056	.000	10.1142	26.1125
Acetone	Methanol	-10.17667*	2.43056	.013	-18.1758	-2.1775
	Ethanol	-9.13000*	2.43056	.024	-17.1292	-1.1308
	1-propanol	-8.98000*	2.43056	.027	-16.9792	-.9808
	Acetonitrile	-18.11333*	2.43056	.000	-26.1125	-10.1142

*. The mean difference is significant at the 0.05 level.

Table S9. Definitive screening design matrix for determining significant factors in Aflatoxin extraction

Factor	Name	Units	SubType	Minimum	Maximum	Mean	Std. Dev.
A	Sample solution volume	mL	Continuous	10.00	20.00	15.00	4.68
B	pH	---	Continuous	5.00	8.00	6.50	1.40
C	Sorbent amount	mg	Continuous	30.00	50.00	40.00	9.35
D	Extraction time	min	Continuous	5.00	10.00	7.50	2.34
E	Desorption solvent volume	µL	Continuous	100.00	300.00	200.00	93.54
F	Desorption time	min	Continuous	10.00	15.00	12.50	2.34
G	NaCl percentage	w/v%	Continuous	0.0000	5.00	2.50	2.34

Standard run	Run	A	B	C	D	E	F	G	ER%
3	1	1	0	1	1	-1	1	-1	84.18
12	2	-1	1	-1	1	1	0	-1	66.68
17	3	0	0	0	0	0	0	0	65.23
4	4	-1	0	-1	-1	1	-1	1	63.14
13	5	1	1	-1	1	-1	-1	0	83.59
11	6	1	-1	1	-1	-1	0	1	63.81
8	7	-1	1	1	0	-1	-1	1	82.32
10	8	-1	-1	1	1	0	-1	-1	61.75
6	9	-1	1	0	-1	-1	1	-1	74.63
15	10	1	1	1	-1	1	-1	-1	58.47
9	11	1	1	-1	-1	0	1	1	67.56
5	12	1	-1	0	1	1	-1	1	58.09
14	13	-1	-1	1	-1	1	1	0	61.27
2	14	0	-1	-1	-1	-1	-1	-1	55.08
16	15	-1	-1	-1	1	-1	1	1	73.12
1	16	0	1	1	1	1	1	1	79.53
7	17	1	-1	-1	0	1	1	-1	65.17

Table S10. ANOVA for determining significant factors in Aflatoxin extraction

Source	Sum of Squares	df	Mean Square	F-value	p-value	significant
**Model**	1162.18	7	166.03	6.06	0.0077	+
A-Sample solution volume	0.2973	1	0.2973	0.0109	0.9193	-
B-pH	396.34	1	396.34	14.47	0.0042	+
C-Sorbent amount	20.62	1	20.62	0.7527	0.4081	-
D-Extraction time	283.32	1	283.32	10.34	0.0106	+
E-Desorption solvent volume	296.06	1	296.06	10.81	0.0094	+
F-Desorption time	132.19	1	132.19	4.83	0.0556	-
G-NaCl percentage	33.36	1	33.36	1.22	0.2984	-
**Residual**	246.52	9	27.39			
**Cor Total**	1408.70	16				

Table S11. ANOVA for determining significant factors or binary interactions in Aflatoxin extraction

Source	Sum of Squares	df	Mean Square	F-value	p-value	Significant
**Model**	4369.52	9	485.50	63.87	< 0.0001	+
A-pH	388.13	1	388.13	51.06	< 0.0001	+
B-Extraction time	749.96	1	749.96	98.66	< 0.0001	+
C-Desorption solvent volume	685.92	1	685.92	90.23	< 0.0001	+
AB	180.50	1	180.50	23.75	0.0006	+
AC	0.6272	1	0.6272	0.0825	0.7798	-
BC	70.09	1	70.09	9.22	0.0125	+
A^2	175.72	1	175.72	23.12	0.0007	+
B^2	247.86	1	247.86	32.61	0.0002	+
C^2	154.84	1	154.84	20.37	0.0011	+
**Residual**	76.02	10	7.60			
Lack of Fit	58.15	5	11.63	3.26	0.1105	-
Pure Error	17.86	5	3.57			
**Cor Total**	4445.54	19				

Table S12. Goodness-of-fit statistics for the quadratic model of aflatoxin extraction recovery.

**Std. Dev.**	2.76		**R^2**	0.9829
**Mean**	80.18		**Adjusted R^2**	0.9675
**C.V. %**	3.44		**Predicted R^2**	0.8596
			**Adeq Precision**	24.3293

Table S13. The optimum condition for the significant factors on the Aflatoxin extraction

Factor	Name	Optimum Level
A	pH	7.41
B	Extraction time	8.17
C	Desorption solvent volume	123.33

Point Prediction

Two-sided Confidence = 95% Population = 99%

Solution 1 of 100 Response	Predicted Mean	Predicted Median	Observed	Std Dev	SE Mean	95% CI low for Mean	95% CI high for Mean	95% TI low for 99% Pop	95% TI high for 99% Pop
ER%	97.6896	97.6896		2.75709	1.17894	95.0628	100.316	84.2392	111.14
